# Dual blockade immunotherapy targeting PD-1/PD-L1 and CTLA-4 in lung cancer

**DOI:** 10.1186/s13045-024-01581-2

**Published:** 2024-07-27

**Authors:** Weishi Cheng, Kai Kang, Ailin Zhao, Yijun Wu

**Affiliations:** 1 Division of Thoracic Tumor Multimodality Treatment, Cancer Center, West China Hospital, Sichuan University, Chengdu, Sichuan China; 2 Peking Union Medical College, Chinese Academy of Medical Sciences, Beijing, China; 3 Laboratory of Clinical Cell Therapy, West China Hospital, Sichuan University, Chengdu, Sichuan China; 4 Department of Hematology, West China Hospital, Sichuan University, Chengdu, Sichuan China

**Keywords:** Lung cancer, Immune checkpoint inhibitor, Bispecific antibody, Combination therapy, Biomarker

## Abstract

Cancer immunotherapies, represented by immune checkpoint inhibitors (ICIs), have reshaped the treatment paradigm for both advanced non-small cell lung cancer and small cell lung cancer. Programmed death receptor-1/programmed death receptor ligand-1 (PD-1/PD-L1) and cytotoxic T lymphocyte-associated antigen-4 (CTLA-4) are some of the most common and promising targets in ICIs. Compared to ICI monotherapy, which occasionally demonstrates treatment resistance and limited efficacy, the dual blockade immunotherapy targeting PD-1/PD-L1 and CTLA-4 operates at different stages of T cell activation with synergistically enhancing immune responses against cancer cells. This emerging dual therapy heralds a new direction for cancer immunotherapy, which, however, may increase the risk of drug-related adverse reactions while improving efficacy. Previous clinical trials have explored combination therapy strategy of anti-PD-1/PD-L1 and anti-CTLA-4 agents in lung cancer, yet its efficacy remains to be unclear with the inevitable incidence of immune-related adverse events. The recent advent of bispecific antibodies has made this sort of dual targeting more feasible, aiming to alleviate toxicity without compromising efficacy. Thus, this review highlights the role of dual blockade immunotherapy targeting PD-1/PD-L1 and CTLA-4 in treating lung cancer, and further elucidates its pre-clinical mechanisms and current advancements in clinical trials. Besides, we also provide novel insights into the potential combinations of dual blockade therapies with other strategies to optimize the future treatment mode for lung cancer.

## Introduction

Lung cancer, originating primarily from the bronchial mucosa or glandular tissues, remains to be one of the most prevalent malignant neoplasms [1]. In 2022, the global incidence of new lung cancer cases was approximately 2.48 million, accounting for 12.4% of all cancer, with the mortality rate from lung cancer comprising 18.7%, thereby marking it as the leading cause of cancer-related deaths [2]. Lung cancer can be histologically categorized into two subtypes: small cell lung cancer (SCLC) and non-small cell lung cancer (NSCLC). NSCLC is the more common subtype, encompassing varieties such as squamous cell carcinoma, adenocarcinoma, and large cell carcinoma. SCLC, on the other hand, is the more aggressive subtype characterized by rapid growth and desperate survival outcome [3].

**Fig. 1:**
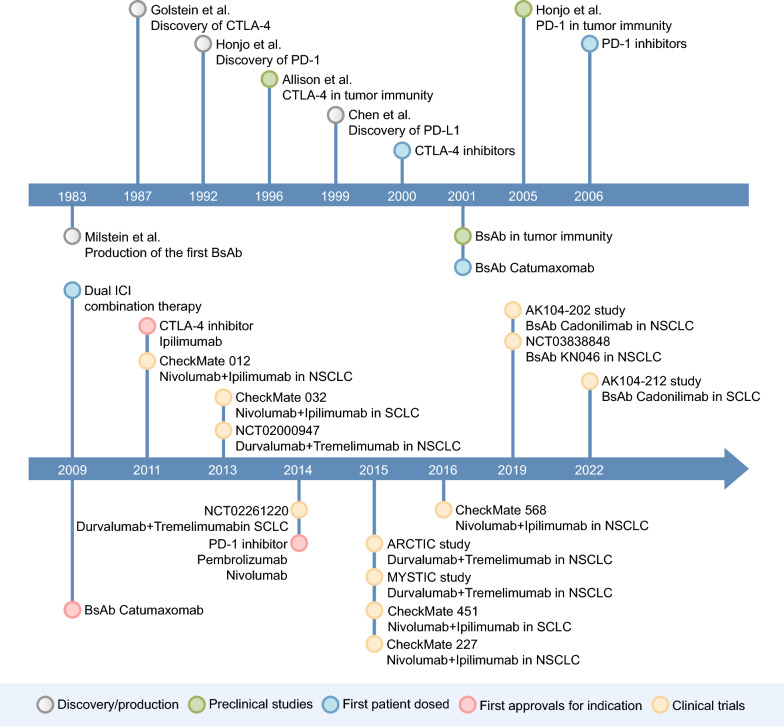
Timing the development of dual ICI combination therapy and bispecific antibodies targeting PD-1/PD-L1 and CTLA-4. Grey dots represent the time of initial discovery or production, green dots represent the time when preclinical studies confirmed its role in tumor immunity, blue dots represent the time when the first patient was dosed, red dots represent the time of the first approvals for indication, and yellow dots represent the time when important clinical trials in lung cancer started. BsAb, bispecific antibodies; CTLA-4, cytotoxic T lymphocyte-associated antigen-4; ICI, immune checkpoint inhibitor; NSCLC, non-small cell lung cancer; PD-1, programmed death receptor-1; PD-L1, programmed death receptor ligand-1; SCLC, small cell lung cancer

In the current landscape of advanced NSCLC treatment, platinum-based doublet chemotherapy remains the standard first-line therapy [4], while in SCLC, the combination of platinum and etoposide is established as the conventional first-line chemotherapy, with topotecan serving as the second-line treatment [5]. Despite its efficacy in the treatment of lung cancer, chemotherapy often fails to halt the progression and recurrence of the disease, subsequently diminishing the survival rates of lung cancer patients [6–8]. In recent years, the advance of immune checkpoint inhibitors (ICIs) has significantly improved the prognosis for lung cancer. These ICIs function by activating anti-tumor immunity to eliminate cancer cells, with programmed death receptor-1/programmed death receptor ligand-1 (PD-1/PD-L1) and cytotoxic T lymphocyte-associated antigen-4 (CTLA-4) being the most common and promising targets, playing a crucial role in the immunotherapy of lung cancer [9, 10]. However, limited efficacy was observed in about 20%-30% of patients with advanced NSCLC receiving ICI monotherapy [11]. And while they show potential efficacy in SCLC, the response rates are relatively low [12]. Therefore, combination immunotherapy approaches based on ICIs, including dual ICI combination therapy and bispecific antibodies (bsAbs), have become one of novel hotspots in the treatment of lung cancer. The history of them, targeting PD-1/PD-L1 and CTLA-4, is summarized in Fig. 1 [13–23].

This review elucidates the application of dual blockade immunotherapy targeting PD-1/PD-L1 and CTLA-4 in lung cancer, providing a comprehensive overview of the utilization and research advancements in dual ICI combination therapy in this context. Furthermore, it will explore the emergence and latest research developments of bsAbs, which promises the expectable future of lung cancer immunotherapy.

## Roles of PD-1/PD-L1 and CTLA-4 in tumor immunity

### Cancer-Immunity Cycle and roles of PD-1/PD-L1 and CTLA-4

The genetic and cellular alterations characteristic of cancer offer pathways for the immune system to generate T-cell responses aimed at identifying and eradicating cancer cells. To enable effective immune-mediated destruction of cancer cells, the Cancer-Immunity Cycle [24] is initiated, striking a delicate balance between non-self recognition and prevention of autoimmunity. Within this cycle, dendritic cells (DCs) capture and process neoantigens that are produced and released by tumors. This process involves signals such as pro-inflammatory cytokines, which are crucial for directing the immune response and avoiding peripheral tolerance to tumor antigens. Following this, the DCs present these antigens to T-cells, thereby triggering and activating effector T-cell responses that are specific to neoantigens. The activated effector T-cells then migrate to the tumor tissue, specifically recognizing and binding to tumor cells through interactions between their T-cell receptors (TCRs) and antigen peptide major histocompatibility complexes (pMHCs), ultimately resulting in the immunological destruction of tumor cells. The death of these tumor cells releases additional neoantigens, thus perpetuating the cycle and further enhancing the response's breadth and depth [24].

However, in cancer patients, the functioning of the Cancer-Immunity Cycle is often suboptimal, due to tumor cells evading immune surveillance by suppressing the activation and effector functions of both the innate and adaptive immune systems [25]. For instance, neoantigens may not be recognized or may be misidentified as self-antigens [24], and the tumor microenvironment (TME) may inhibit the generated effector cells [26]. The advent of cancer immunotherapy has introduced a new paradigm for patients, facilitating the reactivation, amplification, and propagation of the Cancer-Immunity Cycle while minimizing unrestrained autoimmune inflammatory responses. Among these strategies, ICIs have become pivotal in restoring this cycle.

**Fig. 2:**
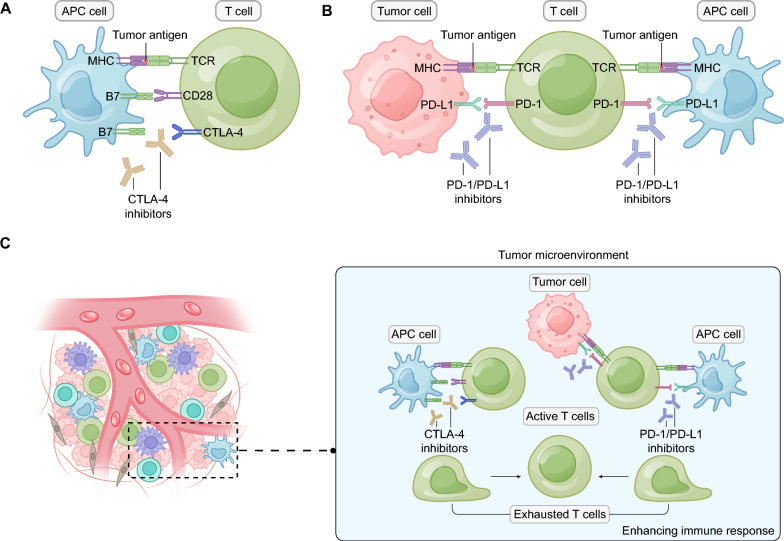
The mechanisms of dual immune checkpoint inhibitor combination therapy in lung cancer. **A** Role of CTLA-4 inhibitors in tumor immunity. CTLA-4 competes with CD28 for binding to B7 (also known as CD80 and CD86) on APC cells, sending inhibitory signals to T cells. CTLA-4 inhibitors can restore immune activity by targeting CTLA-4. **B** Role of PD-1/PD-L1 inhibitors in tumor immunity. PD-1 suppresses the effector T-cells through its ligands PD-L1. PD-1/PD-L1 inhibitors interrupts the PD-1/PD-L1 interaction, thus exerting an anti-tumor effect. **C** The mechanisms of dual immune checkpoint inhibitor combination therapy. Dual inhibitors combination therapy significantly decreases the proportion of exhausted T cells, while increasing active T cells, and has the potential to enhance the immune response. APC, antigen presenting cell; CTLA-4, cytotoxic T lymphocyte-associated antigen-4; MHC, major histocompatibility complex; PD-1, programmed death receptor-1; PD-L1, programmed death receptor ligand-1; TCR, T cell receptor

PD-1/PD-L1 inhibitors and CTLA-4 inhibitors are among the most advanced targets in tumor immune checkpoint therapy, operating at different stages of the immune response. CTLA-4 is expressed at high levels during the initial activation phase of T-cells, where it competes with CD28 for binding to CD80 and CD86 on antigen-presenting cells, sending inhibitory signals to T-cells and preventing their activation and effector functions [27, 28]. In the later stages of the immune response, PD-1 is expressed on activated T-cells. Through its ligands, PD-L1 and PD-L2, it suppresses the effector functions mediated by the T-cell receptor [27, 29]. In cancer immunotherapy, ICIs, by targeting CTLA-4 or interrupting the PD-1/PD-L1 interaction, can restore immune activity, thereby exerting an anti-tumor effect (Fig. 2A, B).

However, a significant challenge in cancer immunotherapy is the resistance to ICI treatment, with immune tolerance playing a critical role. The mechanisms of ICI resistance are categorized into tumor-intrinsic factors and tumor-extrinsic factors [30–32]. Intrinsic resistance arises when tumor cells possess either constitutive or acquired mutations in genes involved in immune regulation, affecting processes such as immune recognition, cell signaling, gene expression, and DNA damage response. Extrinsic resistance are related to TME. TME is composed of tumor-extrinsic factors, including various immune cells, stromal cells, vascular systems, extracellular matrices, and cytokines that influence treatment responses. Through impaired homeostasis between immune suppressive and pro-inflammatory cytokines and mediators, the dynamic interplay in the TME often lead to increased infiltration of myeloid-derived suppressor cells (MDSCs), tumor-associated macrophages, M2 macrophages, and regulatory T cells (Tregs) [31, 33–35]. Immunosuppressive cells and inhibitory cytokines in the TME impair anti-tumor immune responses [36, 37]. For instance, Tregs promote self-tolerance by inhibiting effector T cell (Teff) function through suppressive cytokines and direct contact, thereby limiting inflammation [38, 39]. The infiltration of Tregs into many tumor types has been observed, indicating the presence of an immunosuppressive environment, potentially leading to immunotherapy resistance due to the inability to increase Teffs or reduce Tregs [40–42]. Moreover, continuously evolving immune responses, ranging from antigen presentation to the cytotoxicity of cancer cells and the formation of immune memory, can facilitate evasion of anti-tumor immunity. The inability of T cells to proliferate and diversify may also contribute to ICI resistance [30].

The advent of dual blockade immunotherapy effectively overcomes immune tolerance, enabling bypass of resistance. The clinical benefits of dual blockade immunotherapy are potentially attributed to complementary mechanisms. Given the roles of PD-1/PD-L1 and CTLA-4 in the Cancer-Immunity Cycle, CTLA-4 inhibitors initiates T cell activation, whereas PD-1/PD-L1 inhibitors are involved in the reactivation of effector responses in later stages. Blocking both targets can prevent cancer cells from evading exhausted T-cell immunity while simultaneously delivering signals to antigen presenting cells (APCs) that activate T-cells in the early stages, therefore strengthening the immune response [43, 44]. Furthermore, CTLA-4 inhibitors have been demonstrated to deplete Tregs in the TME and enhance cytotoxic T lymphocyte (CTL)-mediated anti-tumor immunity through enhanced antigen recoginition [45]. Dual blockade immunotherapy also aids in reshaping immune memory, thus facilitating a long-term immune response [46–48]. Additionally, PD-1/PD-L1 inhibitors and CTLA-4 inhibitors exhibit no cross-resistance. Therefore, dual blockade immunotherapy offers substantial advantages over ICI monotherapy.

### Mechanisms of dual immune checkpoint inhibitor combination therapy

Numerous preclinical models have consistently demonstrated that ICIs targeting CTLA-4 and PD-1/PD-L1 can restore the recognition and eradication of tumor cells by T cells. In 1996, Leach et al. [15] demonstrated through murine tumor models that CTLA-4 inhibitors disrupted the signals that typically downregulated endogenous T cell responses, enabling the activation of usually unresponsive T cells. Wong et al. [49], in 2007, examined the effects of PD-1 inhibitors on the ex vivo expansion and functional capacity of human melanoma antigen-specific CD8^+^ CTLs. They found that PD-1 blockade enhanced the frequency and absolute numbers of CTLs, and increased the proportion of antigen-specific CTLs capable of recognizing melanoma targets through degranulation. Furthermore, a kinetic analysis of cytokine secretion revealed that PD-1 blockade also augmented the accumulation of type 1 and type 2 cytokines in the cultures.

Compared to monotherapy with a single ICI, the combination of PD-1/PD-L1 and CTLA-4 inhibitors is more than merely additive. Wei et al. [43, 50] reported that their synergistic effect significantly surpassed the sum of their effects when used individually. The combined use of dual immune checkpoint inhibitors significantly decreased the proportion of exhausted phenotype cytotoxic CD8^+^ T-cells, while simultaneously increasing the presence of active effector T-cells, including active CD8^+^ and CD4^+^ effector cells (Fig. 2C). This shift in the immune cell population from exhausted T cells to active effector cells has the potential to substantially enhance the overall immune response. Sun et al. [51] demonstrated that the combination therapy of PD-1 and CTLA-4 inhibitors significantly reduced the risk of tumor relapse and metastasis in mouse models, thereby markedly prolonging survival (*p* < *0.05*). Similarly, Yeo et al. [52] observed that the use of a single T cell immunoglobulin and ITIM domain (TIGIT) antibody in mouse models did not produce a significant antitumor response, but dual ICI therapy combined with PD-L1 inhibitor Tecentriq elicited a discernible antitumor effect.

Similar findings were reported in the preclinical murine model experiments conducted by Curran et al. [53] In mice pre-implanted with B16-BL6 melanoma, vaccination with B16-Flt-3 ligand (Fvax) combined with a CTLA-4 inhibitor resulted in tumor rejection in 10% of the mice, while adding a PD-1 inhibitor to Fvax increased tumor rejection to 25% of the mice. The combination of CTLA-4 and PD-1 ICIs resulted in tumor rejection in 50% of the mice, more than doubling the efficacy of using either ICI alone. Incorporating a PD-L1 inhibitor into this regimen resulted in tumor rejection in 65% of the mice. Furthermore, the study revealed that the combined blockade of PD-1 and CTLA-4 enhanced the infiltration of Teffs, thereby producing a highly favorable ratio of Teffs to Tregs within the tumor. Additionally, a higher proportion of CTLA-4 and PD-1 positive T cells, which are normally inactivated, remained active.

In view of these findings and other preclinical studies [54], one can infer that the dual ICI therapy combining CTLA-4 with PD-1/PD-L1 is potentially superior to monotherapy with ICIs, providing a solid foundation for subsequent clinical trials.

### Mechanisms of bispecific antibodies

In addition to the dual ICI combination therapy, with the continuous advancements in antibody engineering technology, BsAbs that bind two different epitopes on the same or different antigens offer an alternative approach in cancer therapy for enhancing immunity and reducing toxicity. The concept of merging multiple specific targets into a single antibody dates back to the 1960s [55]. Catumaxomab (anti-CD3 × anti-EpCAM) [21], the first BsAb approved for cancer treatment, heralded the beginning of a new era in cancer therapy with the advent of BsAbs. This innovative approach represents a significant step forward in the ongoing quest to optimize cancer treatments by enhancing both specificity and efficacy.

Natural antibodies consist of paired heavy (H) and light (L) chains, typically exhibiting a Y-shaped configuration. Papain can split them into two functional fragments, specifically the Fab and Fc segments. The Fab fragment comprises the antigen-binding site, while the Fc segment facilitates interactions with cells and effector molecules [56]. Through this mechanism, natural antibodies can participate in a variety of immune processes, including antibody-dependent cell-mediated cytotoxicity (ADCC), antibody-dependent cellular phagocytosis (ADCP), and complement-dependent cytotoxicity (CDC) [57]. However, the two antigen-binding sites of natural antibodies are identical, imparting monospecificity and bivalency characteristics. This structural uniformity restricts the scope of their immune engagement, thus necessitating the exploration of more versatile antibody designs in therapeutic applications.

**Fig. 3:**
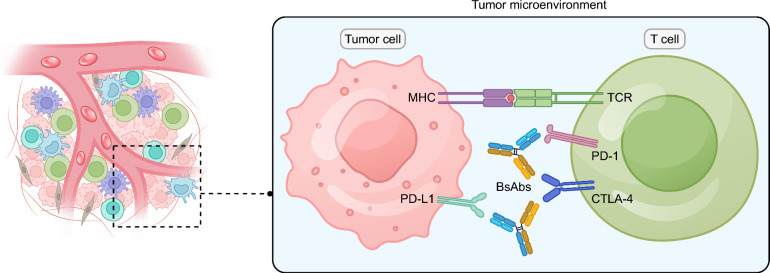
The mechanisms of bispecific antibodies in lung cancer. BsAbs can alleviate the immunosuppressive phenotype by targeting multiple immune inhibitory checkpoints, effectively bypassing the immune tolerance of the tumor microenvironment. BsAb, bispecific antibodies; CTLA-4, cytotoxic T lymphocyte-associated antigen-4; MHC, major histocompatibility complex; PD-1, programmed death receptor-1; PD-L1, programmed death receptor ligand-1; TCR, T cell receptor; TRAEs, treatment-related adverse reactions

The advent of bsAbs has addressed the limitations inherent to natural antibodies. Unlike natural antibodies, bsAbs can be produced only through biochemical or genetic engineering techniques. They consist of two fused single-chain antibodies [58] and can be categorized into two types based on the presence or absence of the Fc fragment: immunoglobulin G (IgG)-like antibodies and non-IgG-like antibodies. The TME is filled with an abundance of immune suppressive cells and ligands, significantly impairing the efficacy of various cancer immunotherapies [59]. BsAbs can alleviate the immunosuppressive phenotype by targeting multiple immune inhibitory checkpoints, effectively bypassing the immune tolerance of the TME [60, 61] (Fig. 3). Furthermore, bsAbs that target co-stimulatory molecules can boost T-cell mediated immune responses [62, 63]. Additionally, bsAbs offer the unique advantage of either cross-linking two types of cells or binding two molecules in cis on the cell membrane. By adjusting the affinity of the two binding sites, bsAbs can help minimize off-target effects in normal tissues and help reduce treatment-related adverse events (TRAEs) [64]. This dual targeting capability not only expands their therapeutic potential but also offers a more targeted approach, reducing collateral damage and improving the specificity of cancer treatments.

Pang et al. [65] performed preclinical experiments on the bsAb Cadonilimab (AK104), targeting PD-1 and CTLA-4. This study showed that Cadonilimab, due to its tetravalent design, exhibits high binding affinity, especially in environments rich in PD-1 and CTLA-4, and can simultaneously bind to distinct cells expressing PD-1 and CTLA-4, demonstrating differential activity compared to ICI. Moreover, Cadonilimab, compared to the combination of anti-PD-1 and anti-CTLA-4, can activate T cells by increasing the secretion of interleukin-2 (IL-2) and interferon-γ (IFN-γ). These characteristics potentially contribute to reducing the toxicity of Cadonilimab and enhancing its antitumor activity. KN046, a bsAb targeting PD-L1 and CTLA-4, was shown in preclinical mouse model trials by Jiang et al. [66] to cause significant increases in the percentage of CD3^+^ CD4^+^ and CD3^+^ CD8^+^ T cells in the tumor and spleen, indicating a robust in vivo immune response. Additionally, residual tumor imaging showed that mice treated with KN046 experienced slower melanoma growth, further comfirming its antitumor efficacy. The outcomes of these preclinical experiments offer crucial evidence for the further advancement of clinical trials involving bsAbs.

## Dual immune checkpoint inhibitor combination therapy

Currently, the dual immunotherapy that combines PD-1/PD-L1 and CTLA-4 inhibitors has received approval for various cancer indications, such as the nivolumab + ipilimumab regimen, a combination of PD-1 and CTLA-4 inhibitors. These inhibitors are extensively applied in the treatment of multiple tumor types, including melanoma [67, 68], renal cell carcinoma [69–72], colorectal cancer [73, 74], hepatocellular carcinoma [75], mesothelioma [76], and esophageal squamous cell carcinoma [77]. Specifically, the PD-L1 inhibitor durvalumab and the CTLA-4 inhibitor tremelimumab are employed in the treatment of hepatocellular carcinoma [78]. In the context of lung cancer, the combination therapy of dual ICIs also holds considerable potential for widespread application.

### Nivolumab in combination with ipilimumab

Nivolumab and ipilimumab, representative drugs of PD-1 and CTLA-4 inhibitors respectively, served as the focus of the CheckMate 012 (NCT01454102) trial [79], a multi-cohort Phase I study. This pivotal study was the first to assess the efficacy and safety of dual ICI combination therapy in patients with advanced NSCLC. Participants in the study were divided into three groups, each receiving one of three distinct dosing regimens: nivolumab 1 mg/kg Q2W (every 2 weeks) + ipilimumab 1 mg/kg Q6W, nivolumab 3 mg/kg Q2W + ipilimumab 1 mg/kg Q12W, nivolumab 3 mg/kg Q2W + ipilimumab 1 mg/kg Q6W. The latter two regimens received particular focus in the trial. In these two cohorts, all treated patients (n = 77) exhibited an objective response rate (ORR) of 43%, significantly higher than the 23% ORR observed in patients treated with nivolumab monotherapy (n = 52). Notably, in subgroups defined by PD-L1 expression levels, the ORR of the dual immunotherapy consistently exceeded that of nivolumab monotherapy. Furthermore, the adverse reactions associated with the dual immunotherapy were tolerable, with grade 3–4 TRAEs at 37% and 33% respectively. The outcomes of this trial suggested that the combination therapy of nivolumab 3 mg/kg Q2W with ipilimumab 1 mg/kg Q12W or Q6W offered more promising clinical outcomes and improved safety profiles compared to previous treatments. This was particularly true for patients with tumor PD-L1 expression of 1% or higher, establishing a solid foundation for subsequent Phase II and III trials.

The Phase II trial CheckMate 568 [80] was designed to evaluate the efficacy and safety of first-line treatment with nivolumab in combination with low-dose ipilimumab in advanced NSCLC patients, and to investigate the correlation between treatment efficacy with PD-L1 expression and tumor mutational burden (TMB). The study enrolled 288 previously untreated advanced NSCLC patients, who received a regimen of nivolumab 3 mg/kg Q2W combined with ipilimumab 1 mg/kg Q6W. Results showed an ORR of 30% across all patients, with an ORR of 41% in patients with PD-L1 expression ≥ 1% and 15% in those with PD-L1 expression < 1%. Regardless of PD-L1 expression levels, patients with a TMB ≥ 10mut/Mb (n = 48: PD-L1 ≥ 1%, 48%; PD-L1 < 1%, 47%) showed benefits in ORR and progressive free survival (PFS) compared to those with TMB < 10 mut/Mb (n = 50: PD-L1 ≥ 1%, 18%; PD-L1 < 1%, 5%). However, no significant correlation was observed between PD-L1 expression and TMB. Grade 3–4 TRAEs were reported in 29% of the patients, with gastrointestinal toxicity being the most common (5%). This trial reaffirmed the efficacy and safety of the combination immunotherapy of nivolumab and ipilimumab as a first-line treatment in advanced NSCLC. It also highlighted TMB as a potential predictive biomarker for the efficacy of dual ICI combination therapy and evaluated its threshold value.

CheckMate 227 [81], an open-label Phase III clinical trial, classified patients with stage IV or recurrent NSCLC based on PD-L1 expression (≥ 1% or < 1%) and assigned them to either part 1a or 1b of the study. Following stratification, patients were randomized into three groups. In part 1a, the treatment options included nivolumab 3 mg/kg Q2W combined with ipilimumab 1 mg/kg Q6W, nivolumab monotherapy, or chemotherapy alone. In part 1b, the treatment regimens included nivolumab 3 mg/kg Q2W combined with ipilimumab 1 mg/kg Q6W, nivolumab plus chemotherapy, or chemotherapy alone. The two-year follow-up results [81] showed that in the part 1a group (patients with PD-L1 expression ≥ 1%), the median overall survival (OS) for the dual ICIs group and the chemotherapy group was 17.1 months and 14.9 months (*p* = 0.007) respectively, with two-year survival rates at 49% and 11%. In the part 1b group (patients with PD-L1 expression < 1%), the median OS for the dual ICIs group was nearly 5 months longer than that of the chemotherapy group (17.2 months vs. 12.2 months, HR = 0.62). Subgroup analysis [82] demonstrated that patients with TMB ≥ 10 mut/Mb receiving dual ICI combination therapy experienced superior ORR and PFS compared to the chemotherapy group (ORR: 45.3% vs. 26.9%, PFS: 7.2 months vs. 5.5 months). This trial further comfirmed that while the efficacy of dual ICI combination therapy was not significantly related to PD-L1 expression, high TMB expression might identify a population that benefits more from the dual ICI combination therapy. Thus, this study has transformed the therapeutic landscape for patients with PD-L1 expression < 1%, marking a new chapter in the dual immunotherapy treatment of advanced NSCLC.

Beyond NSCLC, numerous clinical trials are currently assessing the efficacy and application of dual immunotherapy with nivolumab and ipilimumab in SCLC. CheckMate 032 [83], a multicenter, multi-arm, open-label Phase 1/2 trial, aimed to assess the safety and efficacy of nivolumab monotherapy and nivolumab in combination with ipilimumab dual immunotherapy in SCLC patients who had progressed after one or more previous treatments, including platinum-based chemotherapy. This trial enrolled 216 SCLC patients, treated with either nivolumab 3 mg/kg Q2W (n = 98) as monotherapy or nivolumab combined with ipilimumab in different regimens (1 mg/kg plus 1 mg/kg (n = 3), 1 mg/kg plus 3 mg/kg (n = 61), or 3 mg/kg plus 1 mg/kg (n = 54)) Q3W as dual immunotherapy. The trial results showed that the ORR and median PFS of dual immunotherapy (1 mg/kg plus 1 mg/kg: ORR 33%; 1 mg/kg plus 3 mg/kg: ORR 23%, median PFS 2.6 months; 3 mg/kg plus 1 mg/kg: ORR 19%, median PFS 1.4 months) were generally better than those of nivolumab monotherapy (ORR 10%, median PFS 1.4 months). However, compared to monotherapy, dual immunotherapy also resulted in a higher incidence of grade 3/4 TRAEs. This trial demonstrated the effectiveness and manageable safety of dual immunotherapy in SCLC patients who have failed platinum-based chemotherapy, offering new hope for these patients with limited treatment options.

The double-blind Phase III trial CheckMate 451 [84] enrolled 834 patients with extensive stage SCLC (ES-SCLC) who had not progressed during first-line platinum-based chemotherapy to evaluate the efficacy and safety of dual immunotherapy with nivolumab and ipilimumab. Patients were randomized into three groups, each receiving either nivolumab 1 mg/kg combined with ipilimumab 3 mg/kg Q3W for four cycles, followed by maintenance nivolumab 240 mg Q2W (n = 279), nivolumab monotherapy at 240 mg Q2W (n = 280), or placebo (n = 275). The trial findings showed that, compared to the placebo, neither the dual immunotherapy nor the nivolumab monotherapy significantly improved OS. PFS in the dual immunotherapy group showed a slight improvement over the placebo (1.7 months vs. 1.4 months, HR: 0.72). This study indicated that for SCLC patients who relapse after at least one platinum-based chemotherapy, dual immunotherapy with nivolumab and ipilimumab may be beneficial. However, as a maintenance therapy, it did not achieve the anticipated efficacy.

### Durvalumab in combination with tremelimumab

Concerning the combination of PD-L1 inhibitors and CTLA-4 inhibitors, durvalumab plus tremelimumab represents one of the most notable therapeutic regimens. Durvalumab functions as a PD-L1 monoclonal antibody that blocks the interaction between PD-L1 and PD-1 with high affinity and selectivity [85], while Tremelimumab is a selective human IgG2 monoclonal antibody targeting CTLA-4 [86]. In the Phase 1b study (NCT02000947) [87], the efficacy of durvalumab plus tremelimumab dual immunotherapy in advanced NSCLC patients was assessed for the first time. This study administered escalating dose regimens of durvalumab plus tremelimumab dual immunotherapy in 102 previously untreated advanced NSCLC patients (durvalumab: 3 mg/kg, 10 mg/kg, 15 mg/kg, or 20 mg/kg Q4W, or 10 mg/kg Q2W; and tremelimumab: 1 mg/kg, 3 mg/kg, 10 mg/kg Q4W).The trial determined that most TRAEs were manageable without discontinuing treatment at the 1 mg/kg dose of tremelimumab. As the dose of tremelimumab was gradually increased, a higher frequency of TRAEs was noted, but without an increase in clinical efficacy. Of the 102 patients, 37 (36%) encountered severe TRAEs. The study revealed that durvalumab at 20 mg/kg Q4W in combination with tremelimumab at 1 mg/kg Q4W exhibited manageable tolerability and anti-tumor activity, regardless of PD-L1 expression, laying the groundwork for dosing in Phase III studies.

The Phase III MYSTIC study [88] conducted the initial clinical trial to evaluate the efficacy of durvalumab combined with tremelimumab dual immunotherapy against standard chemotherapy and durvalumab monotherapy in previously untreated advanced NSCLC patients. The study enrolled 1118 previously untreated patients, all of whom had a performance status (PS) of 0–1 and were negative for EGFR/ALK, and who were then randomly assigned to one of three groups: durvalumab 20 mg/kg Q4W as monotherapy, durvalumab 20 mg/kg Q4W in combination with tremelimumab 1 mg/kg Q4W, or platinum-based doublet chemotherapy. The study showed that in patients with PD-L1 expression ≥ 25%, neither of the two immunotherapy achieved the primary endpoint of a significant survival benefit compared to chemotherapy. Furthermore, regardless of PD-L1 expression, the dual ICI combination therapy failed to demonstrate a significant survival benefit in terms of PFS (HR: 1.05, *p* = 0.705) and OS (HR: 0.85, *p* = 0.202) compared to monotherapy and chemotherapy. However, in the subgroup analysis, patients with a bTMB ≥ 20 mut/Mb saw a notable improvement in OS with the dual ICI combination therapy compared to monotherapy and chemotherapy (21.9 months vs. 12.6 months vs. 10 months), identifying TMB as a potential predictive biomarker for the efficacy of durvalumab plus tremelimumab dual immunotherapy.

ARCTIC [89], a Phase III randomized open-label study, assessed the efficacy of durvalumab combined with tremelimumab dual immunotherapy in advanced NSCLC patients who had undergone at least two systemic treatments, including at least one platinum doublet chemotherapy. The trial consisted of two independent substudies based on PD-L1 expression (≥ or < 25%). Substudy B randomly assigned 469 patients with PD-L1 expression < 25% to receive either durvalumab plus tremelimumab dual immunotherapy, monotherapy with either ICI, or standard treatment. The results demonstrated that, compared to chemotherapy, the dual immunotherapy showed some numerical improvement in median OS (11.5 months vs. 8.7 months, HR 0.80, *p* = 0.109) but no difference in median PFS (both 3.5 months, HR 0.77, *p* = 0.056).

In SCLC patients, significant advancements have been made in exploring the combination of durvalumab and tremelimumab as a dual immunotherapy. A Phase I clinical trial [90] assessed the safety and clinical activity of durvalumab plus tremelimumab dual ICI combination therapy in patients with ES-SCLC. In this study, 30 patients were enrolled in an expansion cohort for treatment, initially receiving durvalumab 20 mg/kg Q4W combined with tremelimumab 1 mg/kg Q4W for seven cycles, followed by biannual dosing for two cycles, and then durvalumab 10 mg/kg Q2W for up to 12 months. The trial outcomes demonstrated a median PFS of 1.8 months, a median OS of 7.9 months, and a 12-month OS rate of 41.7%. Seven patients (23%) experienced grade 3/4 TRAEs, with no discontinuations due to TRAEs and no treatment-related deaths were reported. The study validated the promising activity and tolerable safety profile of durvalumab combined with tremelimumab dual immunotherapy in patients with ES-SCLC, setting the stage for further Phase II/III clinical trials. Several ongoing clinical trials are currently investigating durvalumab plus tremelimumab dual ICI combination therapy in SCLC patients (NCT03923270, NCT03043872). Additionally, more ongoing and completed clinical trials of dual ICI combination therapy in NSCLC and SCLC are listed in Tables 1 and 2.

**Table 1:** Ongoing clinical trials of dual immune checkpoint inhibitor combination therapy in NSCLC and SCLC

Targets	Design	Condition	Phase	Primary endpoint	NCT number
**NSCLC**					
PD-1 + CTLA-4	Nivolumab + Ipilimumab + RT	Stage II/III NSCLC	I	Incidence of AEs	NCT04013542
	Nivolumab + Ipilimumab + Oxaliplatin	Advanced NSCLC	I/II	ORR	NCT04043195
	Nivolumab + Ipilimumab + RT vs. Nivolumab + RT	NSCLC with brain metastases	I/II	RP2D; PFS	NCT02696993
	Nivolumab + Ipilimumab + Tocilizumab	Advanced melanoma, NSCLC and UC	II	Incidence of dose limiting toxicity; ≥ G3 AEs	NCT04940299
	Nivolumab + Ipilimumab vs. Nivolumab + Ipilimumab + CT vs. Nivolumab	Stage I-IIIA NSCLC	II	mPR	NCT03158129
	Balstilimab + Botensilimab	Metastatic NSCLC	II	PFS	NCT06322108
	Nivolumab + Ipilimumab vs. Nivolumab + Ipilimumab + LCT	Stage IV NSCLC	III	OS	NCT03391869
	Nivolumab + Ipilimumab vs. Nivolumab + CT vs. CT	Early stage IB-IIIA, operable NSCLC	III	EFS; pCR rate	NCT02998528
	Nivolumab + Ipilimumab vs. Nivolumab	Recurrent stage IV Squamous Cell Lung Cancer	III	OS	NCT02785952
PD-L1 + CTLA-4	Durvalumab + Tremelimumab + Selumetinib	Recurrent/stage IV NSCLC	I/II	MTD, PFS	NCT03581487
	Durvalumab + Tremelimumab vs. Durvalumab + Tremelimumab + RT	Stage IV NSCLC and CC	II	ORR	NCT02888743
	Durvalumab + Tremelimumab + CT	Metastatic NSCLC with EGFR mutation	II	OTRR; OTR	NCT03994393
	Durvalumab + Tremelimumab + CT vs. Durvalumab + CT	Advanced NSCLC, elderly	II	12-month OS	NCT03975114
**SCLC**					
PD-1 + CTLA-4	Nivolumab + BA3071 vs. BA3071	Solid tumor	I/II	dose limiting toxicity; MTD; AEs/SAEs; ORR	NCT05180799
	Nivolumab + Ipilimumab + Plinabulin	Recurrent SCLC	I/II	MTD; PFS	NCT03575793
	Nivolumab + Ipilimumab	LS-SCLC after chemoradiation	II	OS; PFS	NCT02046733
	Nivolumab + Ipilimumab	Recurrent ES-SCLC	II	Change in the ratio of Teff/Treg cells	NCT03670056
PD-L1 + CTLA-4	Durvalumab + Tremelimumab + TRT vs. Durvalumab + Olaparib + TRT vs. Durvalumab + TRT	CR/PR/SD ES-SCLC after CT	I	SAEs; PFS	NCT03923270
	Durvalumab + Tremelimumab + EP vs. Durvalumab + EP vs. EP	Untreated ES-SCLC	III	OS	NCT03043872

AEs: adverse events; CC: colorectal cancer; CR: complete response; CT: chemotherapy; CTLA-4: cytotoxic T lymphocyte-associated antigen-4; EFS: event-free survival; EP: carboplatin or cisplatin + etoposide; ES-SCLC: extensive stage small cell lung cancer; LCT: local consolidative therapy; LS-SCLC: limited stage small cell lung cancer; mPR: major pathologic response; MTD: maximum tolerated dose; NSCLC: non-small cell lung cancer; ORR: overall response rate; OS: overall survival; OTR: objective tumour response; OTRR: objective tumour response rate; pCR: pathologic complete response; PD-1: programmed death receptor-1; PD-L1: programmed death receptor ligand-1; PFS: progression-free survival; PR: partial response; RP2D: recommended phase 2 dose; RT: radiotherapy; SAEs: serious adverse events; SCLC: small cell lung cancer; SD: stable disease; Teff: effector T cell; Tregs: regulatory T cells; TRT: thoracic radiotherapy; UC: urothelial carcinoma

**Table 2:** Completed clinical trials of dual immune checkpoint inhibitor combination therapy in NSCLC and SCLC

Targets	Study design	Patients	Condition	Efficacy		Phase	NCT number
				Median OS, months	Median PFS, months		
**NSCLC**							
PD-1 + CTLA-4	Nivolumab + Ipilimumab	472	Stage IIIB/IV NSCLC	-	5.6/3.9/8.1	I	NCT01454102 (CheckMate 012)
	(p1)Nivolumab + Ipilimumab; (p2) Nivolumab + Ipilimumab + CT	324	Stage IIIB/IV NSCLC	(p1)20.83; (p2)19.35	(p1)5.19; (p2)10.81	II	NCT02659059 (CheckMate 568)
	Nivolumab + Ipilimumab vs. CT	2748	Stage IV/recurrent NSCLC	(p1a)17.1 vs. 14.9; (p1b) 17.2 vs. 12.2	(p1a)5.1 vs. 5.6; (p1b) 5.1 vs. 4.7	III	NCT02477826 (CheckMate 227)
	Nivolumab + Ipilimumab vs. Nivolumab + CT vs. CT	367	Stage IV/recurrent EGFR mutated NSCLC	17.12 vs. 19.35 vs. 15.9	1.54 vs. 5.59 vs. 5.45	III	NCT02864251 (CheckMate 722)
	Nivolumab + Ipilimumab + CT vs. CT	719	Stage IV/recurrent NSCLC	14.13 vs. 10.74	6.83 vs. 4.96	III	NCT03215706 (CheckMate 9LA)
	Nivolumab + Ipilimumab	1041	Stage IV/recurrent NSCLC	1L: 16.76; 2L: 10.45	1L: 5.75; 2L: 3.91	IV	NCT02869789 (CheckMate 817)
PD-L1 + CTLA-4	Durvalumab + Tremelimumab	459	Advanced NSCLC	-	-	Ib	NCT02000947
	Durvalumab + Tremelimumab vs. Durvalumab + Tremelimumab + CT	301	Metastatic NSCLC	14.1 vs. 16.6	3.22 vs. 7.72	II	NCT03057106
	Durvalumab + Tremelimumab vs. Durvalumab vs. SoC vs. Tremelimumab	597	Stage IIIB/IV NSCLC	11.5 vs. 10.0 vs, 8.7 vs. 6.9	3.5 vs. 3.1 vs. 3.5 vs. 2.1	III	NCT02352948 (ARCTIC)
	Durvalumab + Tremelimumab vs. Durvalumab vs. SoC	1118	Stage IV NSCLC	11.9 vs. 16.3 vs. 12.9	3.9 vs. 4.7 vs. 5.4	III	NCT02453282 (MYSTIC)
	Durvalumab + Tremelimumab vs. SoC	953	Stage IV NSCLC	11.7 vs. 9.1	4.2 vs. 5.1	III	NCT02542293 (NEPTUNE)
	Durvalumab + Tremelimumab + SoC vs. Durvalumab + SoC vs. SoC	1186	Stage IV NSCLC	- vs. 13.3 vs. 11.7	6.2 vs.5.5 vs. 4.8	III	NCT03164616 (POSEIDON)
**SCLC**							
PD-1 + CTLA-4	Nivolumab + Ipilimumab + TRT	21	ES-SCLC after CT	11.7	4.5	I/II	NCT03043599
	Nivolumab + Ipilimumab vs. Nivolumab	216	SCLC	7.9/6.0 vs.3.4	2.6/1.4 vs. 1.4	I/II	NCT01928394 (CheckMate 032)
	Quavonlimab + Pembrolizumab	40	ES-SCLC	11.0	2.0	I	NCT03179436
	Nivolumab + Ipilimumab vs. Nivolumab vs. Placebo	907	ES-SCLC	9.17 vs. 10.18 vs. 9.56	1.74 vs. 1.94 vs. 1.41	III	NCT02538666 (CheckMate 451)
PD-L1 + CTLA-4	Durvalumab + Tremelimumab	380	ES-SCLC	7.9	1.8	I	NCT02261220
	Durvalumab + Tremelimumab vs. Durvalumab + Tremelimumab + RT	18	Recurrent SCLC	2.8 vs. 5.7	2.1 vs. 3.3	II	NCT02701400

CT: chemotherapy; CTLA-4: cytotoxic T lymphocyte-associated antigen-4; ES-SCLC: extensive stage small cell lung cancer; OS: overall survival; PD-1: programmed death receptor-1; PD-L1: programmed death receptor ligand-1; PFS: progression-free survival; p1: part 1; p2: part 2; RT: radiotherapy; SCLC: small cell lung cancer; SoC: platinum-based SoC chemotherapy; TRT: thoracic radiotherapy; 1L: first-line; 2L: second-line

### Safety and limitation of dual ICI combination therapy

Despite evidence showing that dual ICI combination therapy significantly enhances therapeutic efficacy compared to ICI monotherapy or chemotherapy alone in various tumor types [67, 69, 74, 81], offering improvements in secondary resistance, prolonged responses, and significant survival benefits, its safety profile and limitations have been the focus of ongoing concern and attention. While the safety of dual ICI combination therapy remains within a tolerable range, several large-scale clinical trials, including CheckMate 9LA [91] and CheckMate 227 [81], have shown that the incidence of grade 3/4 TRAEs with dual ICI combination therapy is comparable to, or even higher than, that of the control chemotherapy group, especially at the grade 3/4 level. The Phase III clinical study CheckMate 9LA [91] demonstrated that the incidence of grade 3/4 TRAEs in the combination therapy group was 47%, higher than in the chemotherapy-alone group (38%). Among these, the dosage of CTLA-4 inhibitors was identified as one of the main factors affecting safety, requiring a delicate balance between dose reduction for safety and dose escalation for efficacy. The most common TRAEs associated with dual ICI combination therapy are dermatological (34% for any grade and 4.2% for ≥ grade 3), endocrine (23.8% and 4.2%), gastrointestinal (18.2% and 2.4%), and hepatic (15.8% and 8.2%) events [91]. Moreover, the population benefiting from dual ICI combination therapy seems limited, with multiple clinical trials [80, 82, 88] suggesting more pronounced survival benefits in populations positive for biomarkers such as TMB and PD-L1, and the range of tumor types benefiting from this approach is relatively narrow. Given the potential for increased TRAE risk with dual ICI combination therapy, the opportunity for combination with other drugs and treatment modalities (such as chemotherapy, targeted therapy, radiation, etc.) remains quite limited, as it may further elevate the risk of TRAEs.

## Bispecific antibodies

**Fig. 4:**
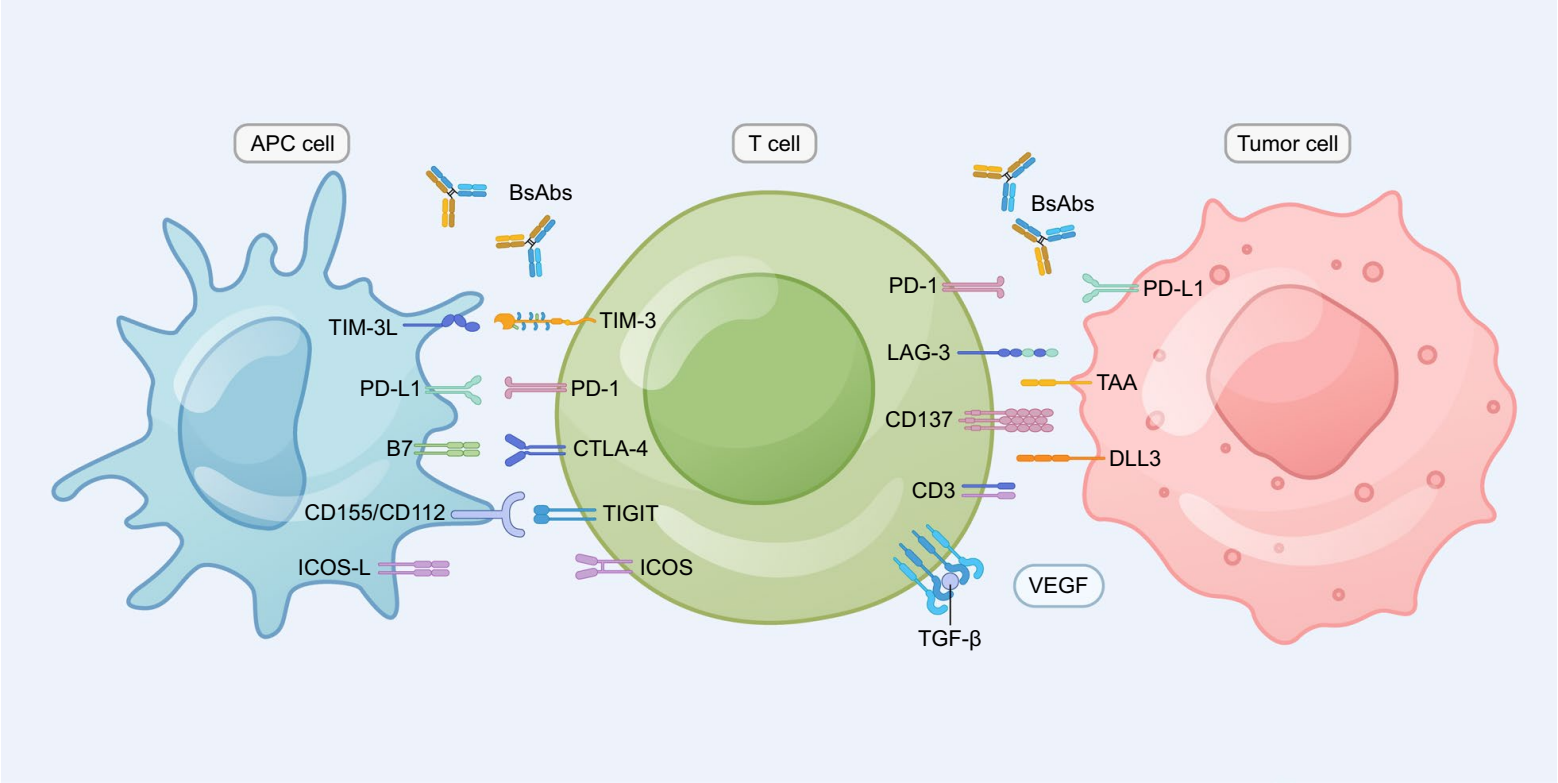
Summary of the various targets for bispecific antibodies in lung cancer. BsAb, bispecific antibodies; CTLA-4, cytotoxic T lymphocyte-associated antigen-4; DLL3, Delta-like ligand 3; ICOS, inducible co-stimulator; LAG-3, lymphocyte activation gene-3; PD-1, programmed death receptor-1; PD-L1, programmed death receptor ligand-1; TAA, tumor-associated antigen; TIGIT, T cell immunoglobulin and ITIM domain; TIM-3, T cell immunoglobulin and mucin-domain containing 3; VEGF, vascular endothelial growth factor

As previous studies have shown [79, 80], monotherapy with ICIs has limited clinical efficacy. In contrast, dual ICI combination therapy, while enhancing survival benefits, also incurs an increased risk of TRAEs and is subject to numerous constraints [91]. The emergence of bsAbs has introduced a novel option for cancer therapy, simultaneously enhancing the efficacy of immunotherapy while reducing treatment-related toxicity. Currently, a variety of bsAbs are employed in the cancer immunotherapy, including blinatumomab (Blincyto) [92], Belantamab mafodotin (Blenrep) [93], teclistamab (Tecvayli) [94], and mosunetuzumab (Lunsumio) [95]. PD-1/PD-L1 and CTLA-4, among the most mature targets within ICIs, are the focus of intense research for their application in lung cancer immunotherapy through bsAbs targeting these checkpoints. This approach offers a novel and potentially more effective method to harness the body's immune response against lung cancer. More various targets for BsAbs in lung cancer is summarized in Fig. 4.

### BsAbs targeting PD-1 × CTLA-4

#### Cadonilimab (AK104)

Cadonilimab, also referred to as AK104, is a humanized bsAb targeting PD-1 and CTLA-4. Functioning as a symmetric tetravalent bifunctional antibody, it binds simultaneously with high affinity to PD-1 and CTLA-4 expressed on tumor-infiltrating lymphocytes (TILs), achieving a co-targeting effect that enhances anti-tumor efficacy while enhancing safety [65, 96]. COMPASSION-01 (NCT03261011) [97] is the first clinical trial to explore the anti-tumor efficacy, tolerability, and safety of cadonilimab in solid tumors, incorporating dose escalation and expansion studies. The study enrolled 119 patients with advanced solid tumors, with 39 in the dose-escalation Phase 1a and 80 in the dose-expansion Phase 1b; mesothelioma was the most common tumor type. The most frequent immune-related adverse event (irAE) observed in the trial was infusion-related reactions, accounting for 18.5%, with overall irAEs and grade 3/4 irAEs occurring in 44.5% and 6.7% of patients, respectively. The study results showed an overall response rate of 13.4% and a median response duration of 12.9 months. This trial confirmed that cadonilimab exhibited promising efficacy and notable tolerability in patients with advanced solid tumors. Furthermore, the study determined a recommended dose of cadonilimab at 6 mg/kg Q2W for subsequent Phase II/III trials.

The Phase 1b/2 COMPASSION-03 (NCT03852251) [98], a multicenter, open-label clinical trial, involved 240 patients with solid tumors. During the dose-escalation phase of the Phase 1b study, patients were administered cadonilimab at either 6 mg/kg or 10 mg/kg Q2W. In the dose-expansion phase, patients were administered cadonilimab at 6 mg/kg or a fixed dose of 450 mg Q2W. In the Phase 2 part of the study, patients with cervical cancer, esophageal squamous cell carcinoma, and hepatocellular carcinoma were treated with cadonilimab at 6 mg/kg Q2W. A total of 67 patients (28%) experienced grade 3/4 irAEs. In the Phase 2 trial, the ORRs for the cohorts with cervical cancer, esophageal squamous cell carcinoma, and hepatocellular carcinoma were 32.3%, 18.2%, and 16.7%, respectively. This trial demonstrated the potential of cadonilimab in treating advanced solid tumors and its manageable safety profile, highlighting its promise as a novel therapeutic option in cancer therapy.

In the context of lung cancer, the AK104-202 study [99], a multicenter, open-label clinical trial, evaluated the efficacy and safety of cadonilimab in treating metastatic NSCLC. The study enrolled 53 patients, dividing them into three groups: Group A consisted of patients who had failed platinum-based doublet chemotherapy and had undergone immunotherapy (IO); Group B comprised patients who had failed platinum-based doublet chemotherapy and demonstrated primary resistance to IO; Group C comprised patients who had failed platinum-based doublet chemotherapy and developed acquired resistance to IO. All patients were administered cadonilimab at a dose of 6 mg/kg Q2W. Regrettably, the study did not meet its primary endpoint, with median OS times of 19.61 months, 4.93 months, and 13.16 months in Groups A, B, and C, respectively. The ORR in Group A was 10%, while no responses were observed in Groups B and C. This trial indicated that cadonilimab exhibits limited efficacy in patients resistant to IO, particularly in those exhibiting primary resistance. The efficacy and safety of cadonilimab in lung cancer remain areas for further exploration. Currently, multiple clinical trials, including AK104-207 (NCT04647344) and AK104-208 (NCT04646330), are ongoing (Table 3). The outcomes of these trials are anticipated to shed light on the role of cadonilimab in lung cancer management and potentially establish new paradigms in treatment regimen.

**Table 3:** Ongoing clinical trials of bispecific antibodies in NSCLC and SCLC

Targets	BsAbs	Combined agent	Condition	Phase	NCT number
**NSCLC**					
PD-1 × CTLA-4	Cadonilimab (AK104)	Anlotinib and docetaxel	Stage IIIB/IIIC/IV NSCLC	I/II	NCT05816499
	Cadonilimab (AK104)	CT	Locally advanced/metastatic NSCLC	I/II	NCT04647344 (AK104-207)
	Cadonilimab (AK104)	Anlotinib	Stage IIIB/IIIC/IV NSCLC	I/II	NCT04646330 (AK104-208)
	Cadonilimab (AK104)	AK119	Advanced solid tumors	I	NCT04572152
	Cadonilimab (AK104)	AK119	Advanced solid tumors	I/II	NCT05559541
	Cadonilimab (AK104)	CT	Stage II/IIIA NSCLC	II	NCT05377658
	Cadonilimab (AK104)	Docetaxel	Locally advanced/metastatic NSCLC	II	NCT05215067
	QL1706 (PSB205)	CT	Stage II/IIIA/IIIB NSCLC	III	NCT05487391
	SI-B003		Recurrent or metastatic solid tumor (Stage IIIB/IV)	I	NCT04606472
	Lorigerlimab (MGD019)	Vobramitamab duocarmazine (MGC018)	Advanced solid tumors	I	NCT05293496
	Lorigerlimab (MGD019)		Squamous cell NSCLC	I	NCT03761017
	MEDI5752	± CT	Advanced solid tumors	I	NCT03530397
PD-L1 × CTLA-4	KN046	Axitinib	Stage IIIB/IIIC/IV NSCLC	II	NCT05420220
PD-1 × PD-L1	IBI318	Lenvatinib	Stage IIIB/IIIC/IV NSCLC, who had failed first-line PD-1/PD-L1 inhibitor therapy	I	NCT04777084
	CTX-8371		Locally advanced/metastatic solid tumors	I	NCT06150664
PD-1 × TIGIT	AZD2936		Stage III/IV NSCLC	I/II	NCT04995523
PD-1 × TIM-3	Lomvastomig		Advanced/metastatic solid tumors	I	NCT03708328
	AZD7789		Stage IIIB/IIIC/IV NSCLC, advanced or metastatic gastric cancer, GEJC	I/II	NCT04931654
	LB1410		Advanced solid tumors, lymphoma	I	NCT05357651
PD-1 × ICOS	XmAb®23104	± Ipilimumab	Advanced solid tumors	I	NCT03752398
PD-1 × VEGF	AK112		Advanced solid tumors	I	NCT04047290
	AK112		Advanced solid tumors	I/II	NCT04597541
	AK112	AK119	Advanced solid tumors	I/II	NCT05689853
	AK112	AK117	Advanced solid tumors	I/II	NCT05229497
	AK112	AK117, ± CT	Advanced malignant tumors	I/II	NCT05214482
	AK112		Stage IIIB/IIIC/IV NSCLC	I/II	NCT04900363
	AK112	CT	Stage IIIB/IIIC/IV NSCLC	II	NCT04736823
	AK112	CT	NSCLC with EGFR mutation	III	NCT05184712
PD-1 × LAG-3	RO7247669		Advanced/metastatic solid tumors	I/II	NCT04140500
	AK129		Advanced malignant tumors	I	NCT05645276
PD-1 × TGF-β	Y101D		Locally advanced/metastatic solid tumors	I	NCT05028556
PD-L1 × VEGF	PM8002	CT	Stage IIIB/IIIC/IV NSCLC	II/III	NCT05756972
PD-L1 × LAG-3	ABL1501		Advanced/metastatic solid tumors	I	NCT05101109
	PS118	Paclitaxel	Advanced solid tumors	I/II	NCT03440437
	IBI323		Advanced solid tumors	I	NCT04916119
PD-L1 × CD47	PF-07257876		NSCLC, SCCHN, Ovarian cancer	I	NCT04881045
	BAT7104		Advanced solid tumors	I	NCT05200013
PD-L1 × Claudin 18.2	Q-1802		Advanced solid tumors	I	NCT04856150
PD-L1 × 4-1BB	ABL503		Advanced/metastatic solid tumors	I	NCT04762641
	FS222		Advanced/metastatic solid tumors	I	NCT04740424
	MCLA-145		Advanced/metastatic solid tumors	I	NCT03922204
	INBRX-105	± Pembrolizumab	Solid tumors	II	NCT03809624
PD-L1 × TIGIT	HLX301		Locally advanced/metastatic solid tumors	I/II	NCT05102214
	HLX301		Locally advanced/metastatic solid tumors, lymphoma	I/II	NCT05390528
**SCLC**					
PD-1 × CTLA-4	Cadonilimab (AK104)	Chiauranib	ES-SCLC	I/II	NCT05505825 (AK104-212)
	Cadonilimab (AK104)	± CT	ES-SCLC	II	NCT05901584
	Cadonilimab (AK104)	+ CT + RT or + vorolanib	ES-SCLC	II	NCT06406673
PD-1 × VEGF	AK112	CT	ES-SCLC	I	NCT05116007
PD-L1 × VEGF	PM8002	CT	SCLC	II	NCT05879068
	PM8002	CT	ES-SCLC	II/III	NCT05844150
PD-L1 × CD47	IMM2520		Advanced solid tumors	I	NCT05780307

CT: chemotherapy; CTLA-4: cytotoxic T lymphocyte-associated antigen-4; ES-SCLC: Extensive Stage Small Cell Lung Cancer; GEJC: gastro-esophageal junction adenocarcinoma; ICOS: inducible co-stimulator; LAG-3: lymphocyte activation gene-3; NSCLC: non-small cell lung cancer; PD-1: programmed death receptor-1; PD-L1: programmed death receptor ligand-1; SCCHN: Squamous Cell Carcinoma of the Head and Neck; TIGIT: T cell immunoglobulin and ITIM domain; TIM-3: T cell immunoglobulin and mucin-domain containing 3; VEGF: vascular endothelial growth factor

#### QL1706

QL1706 (PSB205) is a dual-function bsAb comprising an anti-PD-1 IgG4 and an anti-CTLA-4 IgG1 engineered monoclonal antibody. Furthermore, a novel MabPair technology platform is employed, facilitating the production of two antibodies closely resembling their natural forms from a single host cell and manufactured as a single product [100]. In QL1706, each antibody has undergone individual optimization; for example, the anti-CTLA-4 antibody features a shorter elimination half-life (t_1/2_) to minimize exposure and reduce the risk of irAEs [101]. The first Phase I/Ib clinical trial [101] assessed the efficacy and safety of QL1706 in patients with advanced solid tumors who had failed standard therapy, enrolling 518 patients. The overall ORR was 16.9%, with a median response duration of 11.7 months, including 14.0% in NSCLC and 23.1% in SCLC. The incidences of grade ≥ 3 TRAEs and irAEs were 16.0% and 8.1%, respectively. The trial also identified the maximum tolerated dose (MTD) of QL1706 at 10 mg/kg; following a comprehensive analysis of tolerability, pharmacokinetics/ pharmacodynamics (PK/PD), and efficacy, the recommended Phase 2 dose (RP2D) was established at 5 mg/kg. This clinical trial confirmed the promising anti-tumor activity of QL1706 in solid tumors, particularly in patients with NSCLC, nasopharyngeal carcinoma, and cervical cancer, demonstrating good tolerability. Ongoing randomized Phase II (NCT05576272, NCT05179317) and Phase III (NCT05446883, NCT05487391) trials are in progress, with further results eagerly anticipated (Table 3).

### BsAbs targeting PD-L1 × CTLA-4

KN046 is a novel bsAb targeting PD-L1 and CTLA-4, capable of inhibiting the interactions between PD-L1 and PD-1, as well as between CTLA-4 and CD80/CD86. Preclinical studies [66] have demonstrated that KN046 can mediate the depletion of regulatory T-cells in TME, thereby enhancing anti-tumor immune responses and reducing immunosuppression. A Phase I study of KN046 [102] explored its efficacy, safety, and tolerability in patients with advanced solid tumors. The study enrolled 100 patients, including 59 patients with nasopharyngeal carcinoma and 36 patients with NSCLC. During the dose-escalation phase of the trial, KN046 was administered at doses of 1, 3, 5 mg/kg Q2W, 5 mg/kg Q3W, and 300 mg Q3W, based on a modified toxicity probability interval method. In the dose-expansion phase, the recommended dose was administered. 14.0% of patients experienced grade 3/4 TRAEs, with the most common TRAE being a rash (33.0%). The trial results indicated an overall ORR of 12.5% and a median response duration of 16.6 months. Notably, patients with high expression of CD8 and PD-L1 exhibited a better prognosis. This trial confirmed the promising efficacy and tolerability of KN046 in advanced solid tumors and established a RP2D of 5 mg/kg Q2W for subsequent studies.

The Phase II study (NCT03838848) [103] focused on assessing the efficacy and safety of KN046 in advanced NSCLC patients who had failed or were resistant to platinum-based chemotherapy. The study enrolled 64 patients, divided into Cohort A (n = 30) and Cohort B (n = 34), who received KN046 intravenously at doses of 3 mg/kg and 5 mg/kg, respectively. The results indicated that the ORR for Cohorts A and B were 13.3% and 14.7%, respectively, with a median PFS of 3.68 months for both groups. OS was 19.70 months and 13.04 months for Cohorts A and B, respectively. However, the incidence of grade 3/4 TRAEs was observed at 42.2%. This trial confirmed that both dosing regimens of KN046 exhibited promising efficacy and safety in advanced NSCLC patients who had either failed or were resistant to platinum-based chemotherapy.

### BsAbs targeting other antigens

Beyond the examples previously mentioned, bsAbs, as a pivotal direction in the development of next-generation antibody therapeutics, encompass a wide array of targets under active research and clinical trials. In addition to dual immune checkpoints such as PD-1/PD-L1 and CTLA-4, bsAbs also target co-stimulatory checkpoints like GITR [104, 105], tumor-associated antigens (TAAs) such as EGFR [106, 107], growth factors (GFs) and cytokines including VEGF and TGF-β [108, 109]. This diversity of targets offers a broad and promising research horizon. Specifically for lung cancer patients, numerous clinical trials investigating various bsAbs are currently in progress, as detailed in Tables 3 and 4, exploring their potential to significantly advance the treatment landscape for this challenging disease.

**Table 4:** Completed clinical trials of bispecific antibodies in lung cancer

Targets	BsAbs and conbined agents	Patient numbers	Condition	Efficacy	Phase	NCT number
PD-1 × CTLA-4	Cadonilimab (AK104)	119	Advanced solid tumors	ORR: 13.4%; median DoR: 12.9 months; ≥ G3 irAEs: 6.7%	I	NCT03261011 (COMPASSION-01)
	Cadonilimab (AK104)	338	Advanced solid tumors	≥ G3 irAEs: 28%	I/II	NCT03852251 (COMPASSION-03)
	Cadonilimab (AK104)	53	Advanced/metastatic NSCLC	Median OS (Cohort A-C): 19.61, 4.93, 13.16 months; ORR of Cohort A: 10%	I/II	NCT04172454 (AK104-202)
	QL1706 (PSB205)	518	Advanced solid tumors	≥ G3 irAEs: 8.1%; ORR: 16.9%; median DoR: 11.7 months;	I	NCT04296994; NCT05171790
PD-L1 × CTLA-4	KN046	100	Advanced solid tumors	≥ G3 TRAEs: 14.0%; ORR: 12.5%; median DoR; 16.6 months; median OS: 24.7 months	I	NCT03733951
	KN046	64	NSCLC who failed platinum-based CT	ORR (Cohort A,B): 13.3%, 14.7%; median PFS: both 3.68 months; median OS: 19.70, 13.04 months	II	NCT03838848
PD-1 × VEGF	AK112 + CT	83	Advanced NSCLC	ORR (Cohort 1-3): 53.5%, 68.4%, 40.0%; median PFS (Cohort 2–3): 8.5, 7.5 months	II	NCT04736823

CT: chemotherapy; CTLA-4: cytotoxic T lymphocyte-associated antigen-4; DoR: duration of response; G3: grade 3; irAEs: immune -related adverse events; ORR: overall response rate; OS: overall survival; PD-1: programmed death receptor-1; PD-L1: programmed death receptor ligand-1; TRAEs: treatment-related adverse events; VEGF: vascular endothelial growth factor

CTX-8371, developed by Compass, is a bsAb targeting PD-1 and PD-L1, featuring a 2 + 2 symmetric structure and employing common light chain technology. It functions by converting PD-1 positive cells to PD-1 negative cells through multiple pathways, thereby enhancing anti-tumor efficacy. Its Phase I clinical trial (NCT06150664) is currently in progress in patients with solid tumors, utilizing a 3 + 3 dose-escalation design ranging from 0.1 to 10 mg/kg across five dosage levels. AK112 is a humanized IgG1 bsAb targeting PD-1 and VEGF [110]. Its Phase II trial [109] aimed to evaluate the efficacy and safety of AK112 in combination with chemotherapy in advanced NSCLC patients. The trial results demonstrated that AK112 combined with platinum-based doublet therapy showed promising anti-tumor activity and safety, serving as the first-line treatment in advanced NSCLC patients without driver mutations as well as in patients with EGFR functional mutations who have failed previous EGFR-TKI therapy, and in advanced NSCLC patients who had failed prior systemic platinum-based chemotherapy and PD-1/PD-L1 inhibitor therapy. This provided a valuable potential treatment option for these patients, which was approved for marketing in May 2024. Amivantamab is a bsAb targeting EGFR and MET, with clinical studies indicating significant efficacy in NSCLC patients with EGFR Exon 20 insertions [111]. It has been granted accelerated approval by the Food and Drug Administration (FDA) for second-line treatment in NSCLC with Exon 20 insertions, becoming the first targeted therapy globally for this mutation.

For patients with SCLC, Tarlatamab is a bsAb targeting delta-like ligand 3 (DLL3) and CD3. In 2023, a Phase 2 trial (NCT05060016) [112] involving previously treated SCLC patients indicated that Tarlatamab, administered once every two weeks at doses of 10 mg or 100 mg, exhibited antitumor activity and durable objective responses. Furthermore, it demonstrated favorable survival outcomes with an ORR of 40% in the 10-mg group and 32% in the 100-mg group. The median PFS was 4.9 months and 3.9 months in the 10-mg and 100-mg groups, respectively. And the estimates of OS at 9 months were 68% in the 10-mg group and 66% in the 100-mg group. Besides, this study revealed no new safety concerns compared to the Phase 1 trial. Based on the favorable outcomes of this clinical study, the FDA expeditiously approved Tarlatamab as an innovative therapy for ES-SCLC on May 16, 2024, suitable for patients whose disease continues to progress after platinum-based chemotherapy. This approval also signifies the introduction of the first bsAb targeting DLL3 in the field of SCLC treatment. Additionally, clinical trials are in progress for FPI-2068 targeting EGFR × c-MET (NCT06147037), more bsAbs targeting DLL3 in SCLC [113–115], and others.

## Biomarkers for the efficacy of dual blockade immunotherapy

With the rapid advancement of immunotherapy, the quest for ideal biomarkers to precisely predict therapeutic efficacy and identify the optimal beneficiary population has become increasingly critical. In the context of dual ICI combination therapy, PD-L1 expression and TMB are currently the most researched predictive biomarkers for immunotherapy. The KEYNOTE-024 [116, 117] and KEYNOTE-042 [118] studies suggest that patients with high PD-L1 expression (PD-L1 ≥ 50%) exhibit significantly enhanced antitumor effects and superior survival benefits when treated with pembrolizumab. However, some studies present contrary views, as evidenced in the CheckMate 227 [81] and CheckMate 9LA [119] studies, which showed that both dual ICI therapy and dual ICI combined with chemotherapy achieved more significant survival benefits than chemotherapy alone, regardless of PD-L1 expression. Therefore, for dual ICI combination therapy, PD-L1 expression may not yet serve as a comprehensive and independent biomarker for predicting efficacy. Unlike PD-L1, a protein biomarker, TMB is a genomic biomarker. A higher TMB indicates more neoantigens generated by tumor mutations, leading to increased tumor-infiltrating T lymphocytes and thus enhancing the tumor's immunogenicity and potential immune response against it [120]. Subgroup analysis from the CheckMate 227 study [82] indicated that in patients with high TMB expression (≥ 10 mutations/Mb), the median PFS for the Nivolumab + Ipilimumab group and the chemotherapy group were 7.2 months and 5.5 months, respectively (HR = 0.58, *p* < 0.001), with ORRs of 45.3% and 26.9%, and 1-year PFS rates of 42.6% and 13.2%, respectively, suggesting that high TMB expression may have a predictive role in selecting the population that benefits most from dual ICI combination therapy. Other potential biomarkers include microsatellite instability (MSI) [121–123], mismatch repair deficiency (dMMR) [124–126], a 4-gene inflammatory signature score [127], and the level of infiltration of CD8^+^ T cells or TIL within the tumor [51].

Regarding bsAbs, research on biomarkers remains in its nascent stage. The Phase I trial COMPASSION-01 [97] of the bsAb Cadonilimab, targeting PD-1 and CTLA-4, conducted exploratory analyses on biomarkers. Following the evaluation of the mismatch repair (MMR) status in 54 patients, the study observed that among 10 patients with dMMR status, the confirmed ORR was 50%, with median PFS and OS of 15.5 and 20.5 months, respectively. In contrast, among 44 patients with proficient mismatch repair (pMMR) status, the confirmed ORR was 6.8%, with median PFS and OS of 1.7 and 8.0 months, respectively. Additionally, the study assessed PD-L1 expression in 59 patients, revealing that patients with a PD-L1 Tumor Proportion Score (TPS) ≥ 1 had a higher survival benefit compared to those with PD-L1 TPS < 1 (ORR: 28.6% vs 9.6%; Disease Control Rate (DCR): 85.7% vs 38.5%; median PFS: 7.4 months vs 1.9 months; median OS: 15.2 months vs 8.0 months). In February 2024, Wang et al. [128] conducted an analysis on the gene expression profiles of paired tumor tissues before and after Cadonilimab treatment from 21 patients, aiming to identify biomarkers of clinical response. The results indicated that baseline CD74 gene expression was associated with favorable patient outcomes (OS, HR = 0.33, *p* = 0.0463), and tumors exhibiting high CD74 gene expression at baseline were more likely to present an immunoinflammatory microenvironment. Additionally, high expression of CD74 protein at baseline correlated with better PFS (HR = 0.21, *p* = 0.0065) and OS (HR = 0.35, *p* = 0.0615), highlighting its promising potential as a predictive biomarker for Cadonilimab treatment response.

In the Phase I study of the bsAb KN046 [102], targeting PD-L1 and CTLA-4, exploratory analyses of biomarkers were conducted in 93 patients with advanced solid tumors, evaluating tumor PD-L1 expression. The results indicated that the ORR for patients with PD-L1 expression < 1% and ≥ 1% was 0% and 13.6% (95% CI: 6.4–24.3), respectively. Additionally, patients with positive PD-L1 expression had prolonged median PFS and OS compared to those with negative PD-L1 expression (median PFS: 2.5 months vs 1.3 months; median OS: 19.9 months vs 5.4 months). The study also discovered a correlation between CD8 expression and improved OS. Among combined biomarkers, patients with both PD-L1 positivity and high CD8 expression had better OS compared to other patients (27.1 months vs 9.2 months). In the Phase II trial of KN046 [103], the BIRC-assessed ORR in patients with PD-L1 TC < 1%, 1–49%, and ≥ 50% was 13.9%, 17.6%, and 11.1% respectively, while the median BIRC-assessed PFS was 3.6 months, 3.7 months, and 5.1 months, respectively.

In 2024, Ding et al. [129] conducted a longitudinal plasma proteomic analysis of the bsAb QL1706, which targets both PD-1 and CTLA-4. The study analyzed 113 longitudinal plasma samples from 22 cancer patients treated with QL1706, including six cases of lung cancer. The study revealed that cholesterol metabolism was activated in the disease non-progression (DNP) group and identified the biomarker APOC3, strongly correlated with the partial reconstruction of high-density lipoprotein in the DNP group. The researchers suggested that PA, LDH, and APOC3 could serve as potential biomarkers for predicting the efficacy of QL1706. Additionally, the machine learning model, based on proteomic clinical features, provided accurate predictions for the QL1706 cohort. The predictive capability of this model could also be extended to anti-PD-1 treatment cohorts, thereby laying the foundation for future clinical trials targeting precise immunotherapy responses.

Given the complexity of the immune system and the dynamic and heterogeneous nature of tumors, single biomarkers often fail to predict effectively, necessitating further research into additional biomarkers and clinical prognostic factors. There is anticipation for future studies concerning biomarkers related to bsAbs.

## The combination strategy of dual ICIs and bsAbs

### Combination with chemotherapy

For both NSCLC and SCLC, chemotherapy remains one of the primary first-line treatment methods. It is noteworthy that, during the process of tumor treatment, chemotherapeutic agents exert bidirectional effects on the immune system, causing systemic immune suppression while also eradicating specific immune cells to aid in the reconstruction of a new immune system [130]. On the one hand, drugs such as cyclophosphamide, gemcitabine, and platinum compounds enhance the antigenicity of tumor cells through mechanisms such as calreticulin exposure, autophagy induction, mobility group box 1 protein, and ATP release [131–133]. On the other hand, chemotherapy can increase the sensitivity of tumor cells to immune attacks by enhancing their visibility to the immune system. Moreover, chemotherapeutic agents such as anthracyclines and oxaliplatin interact with DNA replication and repair mechanisms, triggering immunogenic cell death (ICD) and antigen-specific responses [134]. These interactions between chemotherapy and the immune system provide theoretical support for the potential enhanced efficacy of combining chemotherapy with immunotherapy.

In the context of dual ICI combination therapy, numerous clinical trials have explored the clinical efficacy of combining dual ICIs with chemotherapy. The Phase II clinical trial, CheckMate 568 [80], was divided into two parts: the first part treated patients with nivolumab and ipilimumab, while the second part investigated the combination of nivolumab and ipilimumab with chemotherapy. The results indicated that, although the median OS showed minor differences (part 1: 20.83 months, part 2: 19.35 months), the median PFS was significantly prolonged with the combination therapy (part 1: 5.19 months, part 2: 10.81 months). The Phase III study, CheckMate 9LA [119], further examined the clinical efficacy of the combination of nivolumab and ipilimumab with chemotherapy. Patients were randomized into two cohorts: one to receive the combination therapy and the other to receive chemotherapy alone. The findings revealed that the combined treatment led to an extended survival benefit compared to chemotherapy alone (median OS: 14.13 months vs 10.74 months; median PFS: 6.83 months vs 4.96 months). Similar outcomes were observed in the Phase II clinical trial for durvalumab and tremelimumab (NCT03057106) [135], where the cohort treated with durvalumab and tremelimumab combined with chemotherapy demonstrated extended median OS and PFS compared to the dual ICIs treatment cohort (median OS: 16.6 months vs 14.1 months; median PFS: 7.72 months vs 3.22 months). These studies collectively highlight the potential of combining dual ICIs with chemotherapy, with many clinical trials currently ongoing to further explore this combination strategy (NCT03158129, NCT03994393, NCT03975114, NCT03043872).

Research on the combination treatment strategy of bsAbs and chemotherapy is currently in its early exploratory phase. AK112, a bsAb targeting PD-1 and VEGF, has shown promising antitumor activity and safety in its Phase II trial [109]. This efficacy extends to first-line treatment in advanced NSCLC patients without driver mutations, patients with EGFR functional mutations who have failed prior EGFR-TKI therapy, and those who have failed previous systemic platinum-based chemotherapy and PD-1/PD-L1 inhibitor therapy. Numerous clinical trials investigating various bsAbs are actively underway, including Cadonilimab targeting PD-1 and CTLA-4 (NCT04647344, NCT05377658), QL1706 (NCT05487391), and MEDI5752 (NCT03530397). Overall, the prospects for developing combination treatment strategies involving immunotherapy and chemotherapy appear promising.

### Combination with radiotherapy

Besides chemotherapy, radiotherapy is another common first-line treatment method for lung cancer, operating on the principle of directly damaging tumor cells through ionizing radiation. There is substantial evidence indicating that radiotherapy can trigger both local and systemic immune responses through various mechanisms, thereby exerting multifaceted impacts on tumor immunity. For instance, radiotherapy can induce ICD in tumor cells and release damage-associated molecular patterns (DAMPs), which in turn promote the maturation of DCs [136]. These mature DCs can then present tumor antigens to CD8^+^ T cells, initiating specific immune responses [137]. Additionally, radiotherapy can exhibit immunosuppressive characteristics [138–140]. These interactions between radiotherapy and the immune system provide a theoretical basis for combining these two treatment modalities.

The ‘abscopal effect’, referring to the regression and rejection of unirradiated and distant tumor lesions triggered by radiation [141], has been observed in tumors including lung adenocarcinoma [142]. The underlying mechanism for this phenomenon likely involves radiotherapy enhancing the antigen presentation of tumor cells, thereby increasing the production of CD8^+^ T cells. These newly generated T cells are transported through the bloodstream to distant sites, thereby affecting tumors outside the irradiated area [143, 144]. Notably, several studies have demonstrated that immunotherapy can enhance the abscopal effect, while radiotherapy can amplify the efficacy of immunotherapy [140, 145–147]. This interaction provides new avenues and opportunities for combined treatment strategies in immunotherapy.

A randomized Phase II study [148] investigating recurrent SCLC evaluated the efficacy of durvalumab and tremelimumab with or without stereotactic body radiotherapy (SBRT). Eighteen patients were randomly assigned to either Group A, receiving durvalumab and tremelimumab, or Group B, undergoing immunosensitizing SBRT followed by durvalumab and tremelimumab treatment. The results indicated that Group B had an extended median OS compared to Group A (Group A: 2.8 months, Group B: 5.7 months). Numerous clinical trials exploring the combination of dual ICIs with radiotherapy are currently underway, including nivolumab and ipilimumab combined with radiotherapy (NCT04013542, NCT02696993), durvalumab and tremelimumab combined with radiotherapy (NCT02888743, NCT03923270) and others. However, clinical studies investigating the combination of bsAbs with radiotherapy are relatively rare.

### Combination with targeted therapy

Targeted cancer therapy involves the suppression of cancer growth, progression, and metastasis by interfering with specific molecular targets. This strategy includes inhibiting tumor cell proliferation, intervening in the cell cycle, promoting tumor cell differentiation, suppressing metastasis, inducing apoptosis, and obstructing tumor angiogenesis, representing a revolutionary treatment approach [149]. However, targeted therapy currently faces a significant challenge as a substantial number of patients develop resistance to the treatment. Recent studies indicate that targeted cancer therapy can trigger ICD in tumor cells, thereby enhancing the efficacy of ICIs [149]. This discovery has enabled the combination of targeted cancer therapy with immunotherapy, offering a new approach to overcome drug resistance and enhance clinical efficacy.

The Phase I/II MEDIOLA study [150] assessed the efficacy of durvalumab and olaparib combination therapy in patients with metastatic breast cancer harboring germline BRCA1 or BRCA2 mutations. The results demonstrated that after 12 weeks of combination therapy, 80% of the patients exhibited positive disease control and acceptable safety profiles. Additionally, the Phase III clinical trial, IMspire150 study (NCT02908672) [151], assessed the clinical efficacy of vemurafenib and cobimetinib in combination with atezolizumab, an anti-PD-L1 monoclonal antibody, in patients with advanced or metastatic melanoma carrying BRAF V600 mutations. The study outcomes indicated that the combination of targeted therapy with immunotherapy significantly prolonged PFS (15.1 months vs 10.6 months), confirming the feasibility and advantage of the combined treatment approach. Currently, numerous clinical trials exploring the combination of bsAbs and targeted therapies are in progress (NCT05816499, NCT04646330, NCT05420220, NCT04777084).

### Combination with other treatments

Beyond the combined treatment methods mentioned above, many potential combination therapies merit exploration, offering additional possibilities for enhancing the clinical efficacy of immunotherapy. Adoptive immunotherapy using genetically modified T cells expressing antigen-specific chimeric antigen receptors (CARs) is an emerging cancer treatment method with significant promise [152–154]. CARs are synthetic receptors that redirect T cells to tumor surface antigens [155, 156], possessing the unique ability to recognize various cellular targets (including both protein and non-protein entities) and activate T cells without the need for antigen processing and presentation, thereby circumventing human MHC restrictions [157]. Although CAR-T cells can directly eliminate tumor cells, they remain susceptible to inhibition by immune checkpoints. This has brought into focus the strategy of combining CAR-T cell therapy with ICIs. Several preclinical studies [158, 159] using mouse tumor models have validated the feasibility of combining CAR-T cell therapy with ICIs, indicating that ICIs may be an effective strategy to enhance the clinical benefits of CAR-T cell therapy. Clinical trials for this combination therapy are also currently in progress (NCT04003649, NCT03726515).

In the context of SCLC, DLL3 serves as an inhibitory ligand in the Notch signaling pathway and is highly expressed on the surface of SCLC tumor cells, correlating with the progression of SCLC [113, 160, 161]. Rova-T, a humanized monoclonal antibody targeting DLL3, has demonstrated significant antitumor activity in recurrent SCLC, with manageable safety profiles [162]. Within the immune system, DLL3 plays a crucial role in regulating T cell development. Its absence can induce the activity of Notch signaling, which, in conjunction with TCR signaling, promotes T cell differentiation [163]. This suggests that combining Rova-T with immunotherapy is potential for enhancing the clinical benefits against SCLC. Regrettably, a Phase 1/2 study of Rova-T combined with nivolumab ± ipilimumab for treating 2L + ES-SCLC patients (NCT03026166) indicated that, despite its activity in 2L + ES-SCLC, Rova-T with nivolumab and/or ipilimumab were not appropriate due to dose-limiting toxicities (DLTs) [164].

Additionally, various emerging therapeutic modalities, such as CAR-NK cell therapy [165–167] and macrophage-targeted therapy [168, 169], hold the potential for combination with immunotherapy, offering an expanded array of options and possibilities for cancer treatment.

## Discussion and conclusions

Antibodies, protective proteins produced by plasma cells, are capable of specifically binding to antigens. This specificity of antigen binding has propelled the transformation of antibodies into a prominent research area in clinical therapy, leading to the emergence of ICIs [170], which offer new options for cancer treatment. Under normal physiological conditions, immune checkpoints such as PD-1/PD-L1 and CTLA-4 maintain the homeostasis of the immune system, effectively preventing uncontrolled autoimmune responses [171, 172]. However, in cancer patients, these immune checkpoints weaken TCR signaling, leading to reduced effector functions of T-cells and facilitating tumor immune escape [173]. The advent of ICIs presents a potential solution to this dilemma and has demonstrated efficacy in various cancer types, including melanoma, urological cancers, mesothelioma, and others [67, 69, 76].

The current first-line treatment for lung cancer predominantly consists of platinum-based chemotherapy. Studies have indicated that ICI monotherapy offers limited survival benefits, prompting the emergence of combination therapy approaches as a new direction for enhancing anti-tumor efficacy and survival outcomes [11, 12]. Combinations such as nivolumab plus ipilimumab and durvalumab plus tremelimumab in dual ICI combination therapy have effectively increased response rates, albeit potentially raising the risk of TRAEs. With advancements in antibody engineering technology, the development of bsAbs aims to enhance therapeutic efficacy while simultaneously reducing toxicity and the incidence of TRAEs. A variety of novel bsAbs are in the pipeline, poised to enter clinical trials, representing a promising and evolving frontier in the treatment of lung cancer.

As dual blockade immunotherapy, both dual ICI combination therapy and bsAbs have two targets simultaneously, thereby strengthening the tumor immune response and achieving an effect more than merely additive. The efficacy of both therapies significantly surpasses that of monotherapy. However, the dual ICI combination therapy potentially increases the risk of TRAEs while improving response rates. And the introduction of bsAbs has addressed this challenge, achieving a balance of efficacy and safety. In addition, the biological potency and efficacy of bsAbs could potentially be greater than that of the dual ICI combination therapy, which necessitates further research. Regrettably, compared to the detailed study of biomarkers in dual ICI combination therapy, the research on biomarkers related to BsAb treatment remains limited, yet it is advancing rapidly.

However, both dual ICI combination therapy and bsAbs have their limitations. The fixed drug pairing in dual ICI combination therapy and the fixed antibody valency in bsAbs impose numerous constraints on their dosing, efficacy and indications. Moreover, these limitations hinder the development of new treatment strategies, such as combination therapies with chemotherapy and radiotherapy. Additionally, the range of cancers treatable with dual immunotherapy is relatively limited. Even within the same cancer type, significant variation in anti-tumor efficacy among patients can occur due to differences in biomarkers such as PD-L1 and TMB. However, the types and threshold values of these biomarkers still require systematic exploration, especially for bsAbs, where research is comparatively scarce. The management of TRAE risk and the prevention of resistance also pose significant challenges in dual immunotherapy. As the targeted antigens differ across drugs, the anti-tumor action involves activating of multiple signaling pathways, potentially leading to unforeseen immune responses and resistance. Therefore, the interactions between various immune checkpoints, cytokines, and other targets merit further investigation to enhance the effectiveness and safety of these therapies.

Furthermore, the emergence of combination therapies involving chemotherapy, radiotherapy, targeted therapy, and others presents new possibilities and directions for the development of dual ICI immunotherapy and bsAbs. However, these therapies are still in the early stages, and many combination therapies have yet to replicate the outcomes of preclinical studies in clinical trials. The increase in irAEs and healthcare costs, along with the selection of treatment regimens, including dosage, timing, and sequence, remains areas for long-term exploration by researchers.

In summary, despite numerous challenges faced by dual immunotherapy, including dual ICI combination therapy and bsAbs, these approaches retain a promising outlook for future development. It is anticipated that with ongoing advancements in scientific and technological fields, these therapies will continue to be optimized and evolve, heralding a new era in cancer immunotherapy.

## Data Availability

Not applicable.

## Abbreviations

ADCC: Antibody-dependent cell-mediated cytotoxicity
ADCP: Antibody-dependent cellular phagocytosis
AEs: Adverse events
bsAbs: Bispecific antibodies
APC: Antigen presenting cell
CARs: Chimeric antigen receptors
CC: Colorectal cancer
CDC: Complement-dependent cytotoxicity
CR: Complete response
CT: Chemotherapy
CTLs: Cytotoxic T lymphocytes
CTLA-4: Cytotoxic T lymphocyte-associated antigen-4
DAMPs: Damage-associated molecular patterns
DCs: Dendritic cells
DCR: Disease control rate
DLL3: Delta-like ligand 3
dMMR: Mismatch repair deficiency
DNP: Disease non-progression
DoR: Duration of response
EFS: Event-free survival
EP: Carboplatin or cisplatin + etoposide
ES-SCLC: Extensive stage small cell lung cancer
FDA: Food and Drug Administration
Fvax: B16-Flt-3 ligand
GEJC: Gastro-esophageal junction adenocarcinoma
GFs: Growth factors
G3: Grade 3
H: Heavy
ICD: Immunogenic cell death
ICI: Immune checkpoint inhibitor
ICOS: Inducible co-stimulator
IFN-γ: Interferon-γ
IgG: Immunoglobulin G
IL-2: Interleukin-2
IO: Immunotherapy
irAEs: Immune-related adverse events
L: Light
LAG-3: Lymphocyte activation gene-3
LCT: Local consolidative therapy
LS-SCLC: Limited stage small cell lung cancer
MDSCs: Myeloid-derived suppressor cells
MHC: Major histocompatibility complex
MMR: Mismatch repair
mPR: Major pathologic response
MSI: Microsatellite instability
MTD: Maximum tolerated dose
NSCLC: Non-small cell lung cancer
ORR: Overall response rate
OS: Overall survival
OTR: Objective tumour response
OTRR: Objective tumour response rate
QxW: Every x weeks
pCR: Pathologic complete response
PD-1: Programmed death receptor-1
PD-L1: Programmed death receptor ligand-1
PFS: Progression-free survival
PK/PD: Pharmacokinetics/pharmacodynamics
pMHCs: Peptide major histocompatibility complexes
pMMR: Proficient mismatch repair
PS: Performance status
PR: Partial response
p1: Part 1
p2: Part 2
RP2D: Recommended phase 2 dose
RT: Radiotherapy
SAEs: Serious adverse events
SBRT: Stereotactic body radiotherapy
SCCHN: Squamous cell carcinoma of the head and neck
SCLC: Small cell lung cancer
SD: Stable disease
SoC: Platinum-based SoC chemotherapy
TAAs: Tumor-associated antigens
TCRs: T-cell receptors
Teff: Effector T cells
TIGIT: T cell immunoglobulin and ITIM domain
TILs: Tumor-infiltrating lymphocytes
TIM-3: T cell immunoglobulin and mucin-domain containing 3
TMB: Tumor mutational burden
TME: Tumor microenvironment
TPS: Tumor proportion score
TRAEs: Treatment-related adverse events
Tregs: Regulatory T cells
TRT: Thoracic radiotherapy
UC: Urothelial carcinoma
VEGF: Vascular endothelial growth factor
1L: First-line
2L: Second-line
